# Ligase IV syndrome can present with microcephaly and radial ray anomalies similar to Fanconi anaemia plus fatal kidney malformations

**DOI:** 10.1016/j.ejmg.2020.103974

**Published:** 2020-09

**Authors:** Rajesh Madhu, Glenda M. Beaman, Kate E. Chandler, James O'Sullivan, Jill E. Urquhart, Naz Khan, Elizabeth Martindale, Tracy A. Briggs, Jill Clayton-Smith, Jenny Higgs, Gauri Batra, Bronwyn Kerr, Adrian S. Woolf, William G. Newman

**Affiliations:** a Evolution and Genomic Sciences, School of Biological Sciences, Faculty of Biology, Medicine and Health, University of Manchester, Manchester, UK; b Paediatric Neurosciences Department, Alder Hey Children's Hospital NHS Foundation Trust, Liverpool, UK; c Manchester Centre for Genomic Medicine, Manchester University NHS Foundation Trust, Manchester, UK; d Department of Obstetrics and Gynaecology, Royal Blackburn Hospital, Blackburn, UK; e Liverpool Centre for Genomic Medicine, Liverpool Women's Hospital, Liverpool, UK; f Royal Manchester Children's Hospital, Manchester University NHS Foundation Trust, Manchester, UK; g Division of Cell Matrix Biology & Regenerative Medicine, School of Biological Sciences, Faculty of Biology, Medicine and Health, University of Manchester, Manchester, UK

**Keywords:** LIG4, Cystic dysplastic kidneys, Microcephaly, Fanconi anaemia, Radial ray defects, Acrorenal syndrome

## Abstract

Ligase IV (LIG4) syndrome is a rare disorder of DNA damage repair caused by biallelic, pathogenic variants in *LIG4*. This is a phenotypically heterogeneous condition with clinical presentation varying from lymphoreticular malignancies in developmentally normal individuals to significant microcephaly, primordial dwarfism, radiation hypersensitivity, severe combined immunodeficiency and early mortality. Renal defects have only rarely been described as part of the ligase IV disease spectrum.

We identified a consanguineous family where three siblings presenting with antenatal growth retardation, microcephaly, severe renal anomalies and skeletal abnormalities, including radial ray defects. Autozygosity mapping and exome sequencing identified a novel homozygous frameshift variant in *LIG4,* c.597_600delTCAG, p.(Gln200LysfsTer33), which segregated in the family. *LIG4* is encoded by a single exon and so this frameshift variant is predicted to result in a protein truncated by 678 amino acids. This is the shortest predicted LIG4 protein product reported and correlates with the most severe clinical phenotype described to date. We note the clinical overlap with Fanconi anemia and suggest that LIG4 syndrome is considered in the differential diagnosis of this severe developmental disorder.

## 1 Introduction

Ligase IV syndrome (MIM 606593) (Altmann and Gennery, 2016a) is a rare, phenotypically heterogeneous disorder caused by biallelic truncating variants in *LIG4* (O'Driscoll et al., 2001). The clinical features range from increased sensitivity to ionising radiation in a developmentally normal child (Riballo E Doherty et al., 2001) to microcephaly, mild immunodeficiency, developmental delay, and pancytopenia (O'Driscoll et al., 2001; Unal et al., 2009), short stature (Buck et al., 2006; Enders et al., 2006; Murray et al., 2014), severe combined immunodeficiency (SCID) (Buck et al., 2006; Enders et al., 2006), extreme growth failure and syndactyly (Murray et al., 2014).

As LIG4 is encoded by only one exon, and variants that introduce a premature stop codon will result in transcripts that escape nonsense-mediated decay and so produce a truncated protein. The severity of clinical phenotype in ligase IV syndrome has been correlated with the position of the truncating mutation and the size of the resultant truncated protein (Murray et al., 2014). Biallelic truncating mutations at the distal (3′) end of the gene result in a milder phenotype (Murray et al., 2014). Distal mutations *in trans* with a more proximal truncating mutation are associated with a severe growth phenotype, chronic or progressive cytopenia and immune dysfunction. A homozygous 5’ mutation, which removes the entire enzymatic domain, has been reported to cause the most severe phenotype reported to date (Murray et al., 2014).

### 1.1 Clinical report

**Fig. 1:**
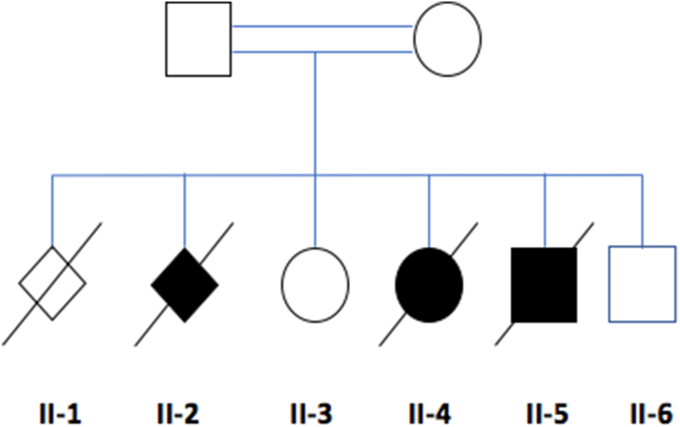
Pedigree of the family affected by LIG4 syndrome, indicating both affected (shaded symbols) and unaffected (unshaded symbols) individuals.

Here, we report a consanguineous British Pakistani family with three affected fetuses each with a severe phenotype resulting in termination of pregnancy (Fig. 1). Key phenotypic features included cystic dysplastic kidneys, oligohydramnios or anhydramnios, microcephaly and intrauterine growth retardation, hypoplastic thumbs and radial ray deformities.

The parents were healthy first cousins and have both had normal renal tract ultrasound scans. The mother had a spontaneous abortion at 8 weeks into her first pregnancy (II-1). She had a termination of her second pregnancy (II-2) at 24 weeks gestation for intrauterine growth retardation, cystic kidneys and anhydramnios, all as visualised on ultrasonography. Post mortem was declined. Her third pregnancy (II- 3) resulted in the birth of a normal female child.

During her fourth pregnancy (II-4), ultrasound scan at 15+ weeks gestation showed severe oligohydramnios and the kidneys were not visualised. A termination of pregnancy was carried out at 16 + 3 weeks’ gestation. The post-mortem examination identified symmetrical growth retardation with all growth parameters >2SD below mean, corresponding to a fetus of 14–15 weeks gestation. Examination of the upper limbs showed bilateral proximally inserted hypoplastic thumbs. The left leg was internally rotated with talipes of the left foot. The right kidney weighed 0.07 g and left kidney weighed 0.08 g, each at the 10th centile (Archie et al., 2006). Histologically the kidneys had cystic dilatation of the tubules surrounded by undifferentiated mesenchyme-like cells, hallmarks of renal dysplasia (Woolf et al., 2004). The urinary bladder and ureters appeared normal on direct examination. External examination of the genitalia was indeterminate but there was histological evidence of primitive ovarian tissue. Genetic investigations revealed a normal female karyotype and a normal chromosomal microarray (OGT ISCA 8 × 60 K oligo array). Chromosome breakage studies were not undertaken. Fetal X ray showed 11 pairs of ribs, but no other evidence of a skeletal dysplasia.

**Fig. 2:**
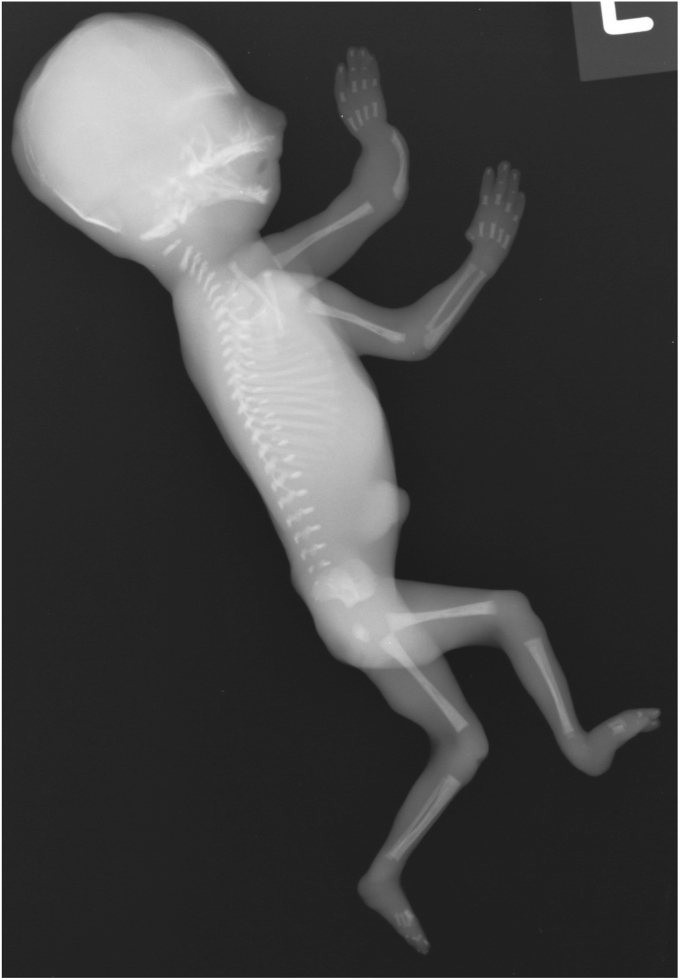
Skeletal survey of Fetus II-5. There are bilateral radial defects of the upper limbs. On the right, the thumb is hypoplastic. The radius and ulna are present. On the left, the radius is absent, the ulna is hypo plastic and bowed and the left thumb is absent.

In the fifth pregnancy (II-5) early ultrasound scans again identified oligohydramnios and abnormal kidneys. On subsequent scans at 17 and 18 weeks the kidneys were not visible. Termination of pregnancy was carried out at 18 + 4 weeks gestation. The post mortem examination identified a male fetus with symmetrical intrauterine growth retardation, with all external growth parameters measuring <5th centile for gestation. The ears were low set and eyelids fused. Upper limb examination showed a small left forearm with the hand connected to the elbow on the left. The left thumb was absent and the right thumb was hypoplastic. There was bilateral positional talipes of the lower limbs, with the lower limb smaller on the left than the right and left 2–3 toe syndactyly. External male genitalia were seen but testes were absent. A skeletal survey confirmed the absent left thumb and radius, hypoplastic and bowed left ulna, hypoplastic right thumb and a hypoplastic sacrum (Fig. 2). On direct inspection both kidneys appeared to be absent with a single small nodule within the pelvis weighing 0.08 g, representing a possible developing pelvic kidney. Histology confirmed that the nodule contained renal tissue with glomeruli and both proximal and distal tubules, and no cystic changes. The ureters were not visualised and a bladder with a thin fibrous wall was present. Cytogenetic analysis revealed a normal male karyotype. Chromosome breakage studies were requested, but cell cultures failed to grow.

The mother gave birth to a normal boy following her sixth pregnancy (II-6).

The couple provided informed consent for participation in a study approved by South Manchester Ethics committee (11/H1003/3, IRAS 64321) and the University of Manchester to determine the cause of their affected children.

Autozygosity mapping using Affymetrix Genome-Wide SNP6.0 array was performed on samples from the healthy girl and boy (II-3 and II-6) and the fetal samples from affected fourth and fifth pregnancies (II-4 and II-5). AutoSNPa analysis revealed two shared regions of homozygosity >2 Mb between the two affected individuals, totalling over 9.8 Mb (Carr et al., 2006), on chromosome 13 (107,914,927–112,790,278) and chromosome 17 (4,323,988–9,272,820). Exome sequencing was undertaken on a sample from II-5, using Agilent SureSelect v5 enrichment. A total of 25,369 variants were identified in the exome data. Filtering to remove those with a frequency of >1% within the Exome Variant Server (~6,500 individuals), Exome Aggregation Consortium (>60,000 individuals), 1000 Genome databases or those seen previously within an in-house dataset of over 600 individuals refined to four homozygous rare variants in the regions of homozygosity (Supplementary Table 1). Of these four variants, a homozygous novel four base pair deletion in *LIG4* c.597_600delTCAG was predicted to result in a pathogenic frameshift variant p.(Gln200LysfsTer33) and was the most credible variant to account for the phenotype. No homozygous loss of function variants (frameshift or nonsense) in *LIG4* are present in the gnomAD database (Karczewski et al., 2020) of >140,000 individuals.

Sanger sequencing confirmed the presence of the homozygous variant in the affected siblings; both parents (I-1 and I-2) and the unaffected siblings (II-3 and II-6) were heterozygous for this variant.

As tiny dysplastic kidneys can be caused by biallelic variants in *FRAS1* (McGregor et al., 2003) or *FREM2* (Jadeja et al., 2005); X linked Kallman syndrome due to *KAL1* variants (Deeb et al., 2001), and multicystic dysplastic kidneys by heterozygous *HNF1B* variants (Clissold et al., 2015), we noted that there were no pathogenic variants in these genes. Furthermore, targeted analysis of the many genes that result in the clinically and genetically heterogeneous Fanconi anemia (FANCA-FANCW) revealed no putative pathogenic variants (Mehta et al., 1993–2020).

## 2 Discussion

We report a severe phenotype of LIG4 syndrome due to a biallelic truncating mutation p.(Gln200LysfsTer33) in *LIG4*. This variant is the most proximal pathogenic variant reported to date (Murray et al., 2014). Previous studies indicated that truncating mutations close to the N-terminus result in a near complete loss of ligase 4 enzyme function and correlate with a more severe phenotype (Murray et al., 2014). The data presented here is consistent with this hypothesis. We speculate that variants that occur more proximally than the one reported here will also be incompatible with life, as is seen with complete knockout of *lig4* in mice (Barnes et al., 1998).

Microcephaly and severe intrauterine growth retardation (IUGR) have both been previously reported in severe cases of LIG4 syndrome (Murray et al., 2014). However, severe renal anomalies, as seen in these affected fetuses, are not a common feature of LIG4 syndrome. In a case series of eleven children with LIG4 syndrome, renal anomalies including atrophic or dysplastic kidneys were reported in only two cases, although renal imaging was not reported for all cases (Murray et al., 2014). One male with biallelic variants in *LIG4* presented with dysplastic kidneys, bilateral vesicourethric reflux and urethral valves (IJspeert et al., 2013), whereas the other was a female with an ‘atrophic’ kidney (Murray et al., 2014). The sibling of the latter case, who also had LIG4 syndrome, did not have documented renal involvement. In contrast to the severe renal involvement affecting all of the fetuses in the family described here, the milder renal tract anomalies did not appear to segregate in the other families with the specific *LIG4* variants. Renal imaging would be appropriate in all individuals with LIG4 syndrome to establish the relationships between *LIG4* variants and renal tract disease.

Of note the position of the variant within *LIG4* did not correlate with the occurrence of milder kidney or lower urinary tract involvement. It will be informative to document renal involvement in other cases with proximal truncating *LIG4* variants. In an individual with biallelic variants in *LRIG2*, the causal gene for urofacial syndrome (MIM 615112), and a homozygous *LIG4* missense variant, the affected child had recurrent episodes of urosepsis, secondary to severe vesicoureteral reflux, leading to left kidney hypoplasia and scarring (Fadda et al., 2016). In this case the lower urinary tract disease and kidney disease was attributed to co-occurrence of urofacial syndrome which is characterised by abnormal bladder voiding and vesicoureteric reflux.

Murine *lig4* variant models have not been reported to have renal or urinary tract involvement (Rucci et al., 2010), but there is no specific description of whether the kidneys were examined. Further homozygous null mutants die in midgestation at a time before the metanephric kidney initiates (Barnes et al., 1998; Frank et al., 1998). Of note, the GUDMAP mouse gene expression database (Gudmap.org.Genito) shows that *LIG4* is highly expressed in the ureteric bud and metanephric mesenchyme. Indeed, it has been postulated that aberrant induction between these respective precursors of collecting ducts and nephrons underlies the pathogenesis of renal dysplasia (Woolf et al., 2004).

Radial ray anomalies have not been reported in individuals with LIG4 syndrome. Previous case reports show other skeletal abnormalities, including hypoplastic ribs, fusion of carpal bones or abnormal vertebrae (Murray et al., 2014). The radial ray defects in combination with IUGR, microcephaly and renal defects suggest an overlapping phenotype between LIG4 syndrome and Fanconi anaemia (FA, MIM 227650), a clinically and genetically heterogeneous autosomal recessive disorder which like LIG4 syndrome impacts on the DNA damage repair pathway, and can present with multiple congenital anomalies, including lethal antenatal presentation (Table 1). Approximately half of all patients with FA have renal involvement, including dysplastic, pelvic, malrotated kidneys, crossed fused ectopia, horseshoe, and multicystic kidneys (Sathyanarayana et al., 2018).

**Table 1:** Comparison of clinical features in individuals with LIG4 syndrome and Fanconi anaemia.

	LIG4 syndrome (Murray et al., 2014)^,^ (Altmann and Gennery, 2016a)	Fanconi Anaemia (Mehta et al., 1993–2020)^,^ (Tischkowitz and Hodgson, 2003)^,^ (Dokal, 2000)
IUGR	Common	Common
Short stature	Common	Variable
Microcephaly	Common	Common
Upper limb thumb/radial ray defects	Rare (this report)	35%
Craniofacial malformation	Common, beak-like nose, prominent mid-face, receding forehead and micrognathia	(Altmann and Gennery, 2016a)Rare
Skeletal malformation	Not reported	70%
Renal malformation	Rare	20–34%
Skin pigmentation (café au lait macules)	Reported	40–64%
Intellectual and/or developmental delay	Variable	(O'Driscoll et al., 2001)10–16%
Ophthalmological defect (microphthalmia)	Not reported	20–38%
Cardiac defect	Not reported	6–13%
Gastrointestinal tract defect	Not reported	5–16%
Hearing loss	Not reported	10%
Haematological defect (pancytopenia)	Common	90%
Immunological defect	Common, prone to infection	Reported
Acute myeloid leukaemia	Not reported	Common (500 fold increased risk)
Solid tumour	Not reported	Common, especially head and neck squamous cell carcinoma
Chromosome instability	Not reported	Yes
Cellular radiosensitivity	Yes	Reported

Renal anomalies and limb defects are characteristic of a clinically heterogeneous group of disorders termed acrorenal syndrome (Kroes et al., 2004). In a series of 197 cases with acrorenal syndrome, eight had radial ray defects and renal anomalies without a syndromal diagnosis (Murray et al., 2014). Our report suggests that LIG4 syndrome should be a differential diagnosis to consider in individuals with acrorenal syndrome.

This report expands the phenotypic spectrum LIG4 syndrome due to premature truncating mutations to include antenatal severe renal phenotype and radial ray skeletal anomalies. The finding in this family facilitates the option for prenatal testing in future pregnancies, and cascade genetic testing in the wider family if married consanguineously to determine their reproductive risks. We recommend that *LIG4* is added to the extensive list of genes to be tested when a diagnosis of Fanconi anaemia is considered as part of the differential diagnosis.

## CRediT authorship contribution statement

**Rajesh Madhu:** Methodology, Formal analysis, Investigation, Writing - original draft. **Glenda M. Beaman:** Methodology, Formal analysis, Investigation, Writing - review & editing. **Kate E. Chandler:** Resources, Writing - review & editing. **James O'Sullivan:** Methodology, Formal analysis, Investigation, Writing - review & editing. **Jill E. Urquhart:** Methodology, Formal analysis, Investigation, Writing - review & editing. **Naz Khan:** Resources, Writing - review & editing. **Elizabeth Martindale:** Resources, Writing - review & editing. **Tracy A. Briggs:** Resources, Writing - review & editing. **Jill Clayton-Smith:** Resources, Writing - review & editing. **Jenny Higgs:** Resources, Writing - review & editing. **Gauri Batra:** Resources, Writing - review & editing. **Bronwyn Kerr:** Resources, Writing - review & editing. **Adrian S. Woolf:** Conceptualization, Methodology, Writing - original draft, Supervision, Project administration, Funding acquisition. **William G. Newman:** Conceptualization, Methodology, Writing - original draft, Supervision, Project administration, Funding acquisition.
