# Supplementary material for: Ligase IV syndrome can present with microcephaly and radial ray anomalies similar to Fanconi anaemia plus fatal kidney malformations
Source: Eur J Med Genet. 2020 Sep;63(9):103974. doi: 10.1016/j.ejmg.2020.103974 (PMC7445424; doi:10.1016/j.ejmg.2020.103974)
Supplement: Multimedia component 1 [file mmc1.docx]

Homozygous variants of interest in those regions:

| Chr | St | End | Gene | Transcript | Consequence | cDNA | Protein | Rs number | gnomAD | Prediction to be damaging |
| --- | --- | --- | --- | --- | --- | --- | --- | --- | --- | --- |
| 13 | 108863017 | 108863020 | LIG4 | ENST00000442234 | frameshift | c.597_600delTCAG | p.(Gln200LysfsTer33) | Rs752339466 | Hom 0/250162  Het 4/250162 | - |
| 17 | 4607372 | 4607372 | PELP1 | ENST00000572293 | missense | c.185G>C | p.(Gly62Ala) | Rs570490093 | Hom 3/265104  Het 430/265104 | Polyphen- unknown  Sift - deleterious |
| 17 | 8661610 | 8661610 | SPDYE4 | ENST00000582989 | missense:  NMD_transcript | c.91G>A | p.(Glu31Lys) | Rs192464033 | Hom 0/184820  Het 138/184820 | Polyphen- unknown  Sift - deleterious |
| 17 | 8661684 | 8661684 | SPDYE4 | ENST00000582989 | missense:  NMD_transcript | c.17C>T | p.(Ala6Val) | Rs34694079 | Hom 7/188518  Het 1041/188518 | Polyphen -unknown  Sift - tolerated |
